# Sediment and nutrient storage in a beaver engineered wetland

**DOI:** 10.1002/esp.4398

**Published:** 2018-05-16

**Authors:** Alan Puttock, Hugh A. Graham, Donna Carless, Richard E. Brazier

**Affiliations:** ^1^ Geography University of Exeter Exeter UK EX1 2EG

**Keywords:** Eurasian beaver, ecosystem engineering, sediment storage, nutrient storage, soil erosion

## Abstract

Beavers, primarily through the building of dams, can deliver significant geomorphic modifications and result in changes to nutrient and sediment fluxes. Research is required to understand the implications and possible benefits of widespread beaver reintroduction across Europe. This study surveyed sediment depth, extent and carbon/nitrogen content in a sequence of beaver pond and dam structures in South West England, where a pair of Eurasian beavers (Castor fiber) were introduced to a controlled 1.8 ha site in 2011. Results showed that the 13 beaver ponds subsequently created hold a total of 101.53 ± 16.24 t of sediment, equating to a normalised average of 71.40 ± 39.65 kg m^2^. The ponds also hold 15.90 ± 2.50 t of carbon and 0.91 ± 0.15 t of nitrogen within the accumulated pond sediment.

The size of beaver pond appeared to be the main control over sediment storage, with larger ponds holding a greater mass of sediment per unit area. Furthermore, position within the site appeared to play a role with the upper‐middle ponds, nearest to the intensively‐farmed headwaters of the catchment, holding a greater amount of sediment. Carbon and nitrogen concentrations in ponds showed no clear trends, but were significantly higher than in stream bed sediment upstream of the site.

We estimate that >70% of sediment in the ponds is sourced from the intensively managed grassland catchment upstream, with the remainder from *in situ* redistribution by beaver activity. While further research is required into the long‐term storage and nutrient cycling within beaver ponds, results indicate that beaver ponds may help to mitigate the negative off‐site impacts of accelerated soil erosion and diffuse pollution from agriculturally dominated landscapes such as the intensively managed grassland in this study. © 2018 The Authors. Earth Surface Processes and Landforms published by John Wiley & Sons Ltd.

## Introduction

In the UK intensively managed grasslands, soil erosion rates of between 0.5 and 1.2 t ha^‐1^ yr^‐1^ have been reported (Bilotta *et al*., 2010; Gregory *et al*., 2015), and agricultural erosion rates can exceed 140 t ha^‐1^ yr^‐1^ (Chambers and Garwood, 2006). Such rates exceed typical soil formation rates of 0.1 t ha^‐1^ yr^‐1^ under intensive land use (Verheijen *et al*., 2009), which constitutes a net soil loss (Montgomery, 2007). In 2009, the cost of soil erosion in the UK was estimated at £45 million per annum, much of which was due to the off‐site impacts associated with sediment and nutrient pollution (DEFRA, 2009). To manage the environmental problems faced in the landscape there is an increasing interest in ‘working with natural processes’ (Environment Agency, 2017) one such option in the UK is the reintroduction of the Eurasian beaver (Castor fiber).

Beavers are often termed ecosystem engineers (Jones *et al*., 1994). They can extensively modify riparian and river systems to create habitats more suitable for habitation (McKinstry *et al*., 2001; Nyssen *et al*., 2011; Nummi and Holopainen, 2014). The most significant geomorphic impact of beavers results from their dam building ability and the consequent impoundment of large volumes of water and potentially associated sediment and nutrient accumulation in ponds (Naiman *et al*., 1988; Butler and Malanson, 2005; Hood and Bayley, 2008). Dam and pond features can alter hydrological regimes, both locally and downstream (Polvi and Wohl, 2012; Burchsted and Daniels, 2014). The resulting increased structural heterogeneity of the environment (Rolauffs *et al*., 2001) also creates a diverse range of habitats (Rosell *et al*., 2005) with an increasingly recognised potential as a habitat restoration tool (Law *et al*., 2017). In addition to increasing biodiversity (Law *et al*., 2017), it has been suggested that, due to their engineering activity, beavers could play a role in the management of river catchments (Puttock *et al*., 2017).

Beaver damming can cause major changes in landscape connectivity to occur; increasing water storage on floodplains and reconnecting floodplains with channels (Macfarlane *et al*., 2015). Beaver dams can also reduce channel flow velocity (Burchsted and Daniels, 2014) and attenuate storm event hydrographs (Nyssen *et al*., 2011) with positive impacts on flood risk alleviation, attributed to the increased storage capacity (Collen and Gibson, 2000) and reduced downstream connectivity (Puttock *et al*., 2017). Beaver pond–dam complexes have been reported to act as sediment traps, due to the rapid decrease in velocity when water enters a pond (Butler and Malanson, 1995; Klotz, 2007). The altered flow regimes also modify nutrient and chemical cycling in ponds and rivers which, combined with trapping and storage of sediment, can impact upon downstream water quality (Naiman *et al*., 1986; Dillon *et al*., 1991).

Previous research by the authors, monitoring water quality above and below a sequence of beaver dams, found a reduction in downstream concentrations and loads of nitrogen, phosphate and suspended sediment during storm flows (Puttock *et al*., 2017). The work highlighted the role that beaver reintroduction might play in managing degraded agricultural landscapes. Another recent study of beaver activity in UK agricultural landscapes has shown similar downstream reductions in nitrogen and phosphorus concentrations (Law *et al*., 2016).

The extent to which beavers alter river systems depends on habitat suitability, population numbers and catchment characteristics (Butler and Malanson, 2005). By promoting deposition, beaver dams can lead to the infilling of beaver ponds with sediment which, over time, can be colonised and stabilised by vegetation and are referred to as beaver meadows (Naiman *et al*., 1988; Burchsted and Daniels, 2014; Johnston, 2014). As such, sediment storage has been shown to increase with beaver pond age (Gurnell, 1998). However, it must also be recognised that this beaver meadow end state is not reached in all situations and beaver dams can fail (Butler and Malanson, 2005). Typically during high energy rain events (Klimenko and Eponchintseva, 2015) beaver dam failure can result in releases of sediment (Polvi and Wohl, 2012, de Visscher *et al*., 2014) meaning that sediment storage in ponds can be transient (Levine and Meyer, 2014).

The combined impact of a beaver dam sequence on flow dynamics results in a change in deposition and storage dynamics downstream through a sequence of ponds. Furthermore, while it has been identified that beaver dams can store large amounts of sediment (Lamsodis and Ulevičius, 2012), it has also been shown that beaver activity (i.e. burrowing) can remobilise sediment (Butler and Malanson, 1995) and that in‐pond erosion can occur and constitute a source (de Visscher *et al*., 2014). As such, it cannot be assumed that all sediment within a beaver pond sequence originates from upstream and therefore sediment source must also be considered.

Eurasian beavers were once widespread across Europe (Halley and Rosell, 2002). However, populations were greatly reduced by human activities (Collen and Gibson, 2000) with beaver being effectively absent from the UK by the 16th century (Conroy and Kitchener, 1996). Recent reintroduction programs have seen the re‐establishment of colonies across much of their previous European geographical range (de Visscher *et al*., 2014). Yet, due in part to the contemporary absence from European countries, most existing research has focused on the North American beaver (Castor canadensis), rather than the Eurasian beaver (Castor fiber). Perhaps more importantly, North American research has been undertaken across very different landscapes to the intensively‐farmed land that is typical of Europe and, with notable exceptions (Stefan and Klein, 2004, de Visscher *et al*., 2014), is understudied in Europe (Puttock *et al*., 2017). European landscapes are characterised by a long history of intensive agriculture, high human population density and dense networks of infrastructure (Brown *et al*., 2018) meaning beaver impacts cannot be presumed directly comparable with North American studies (Gurnell, 1998). As a consequence, further understanding of how beavers impact on the environment is required. Such information will inform policy regarding both their reintroduction into countries like the United Kingdom and the wider management of these animals across Europe.

The aim of this paper is to present results from a controlled monitoring experiment to improve understanding of the impacts of the Eurasian beaver on sediment and nutrient storage within intensively managed agricultural landscapes. To meet this aim, the study addresses the following hypotheses:

Hypothesis 1 (Sediment and nutrient storage) Individual beaver ponds create significant sediment and nutrient stores, in excess of local channel storage.

Hypothesis 2 (Storage downstream) In a sequence of beaver ponds, in‐pond sediment and associated nutrient storage significantly changes downstream.

Hypothesis 3 (Storage and age) Sediment and nutrient storage in beaver ponds is positively correlated with age as older ponds accumulate more sediment over time.

Hypothesis 4 (Sediment source) Sediment and nutrients stored in ponds is sourced from both in‐site redistribution by beaver activity and sediment eroded from intensively managed grassland upstream, but is dominated by the latter.

## Methods

### Study site

**Figure 1:**
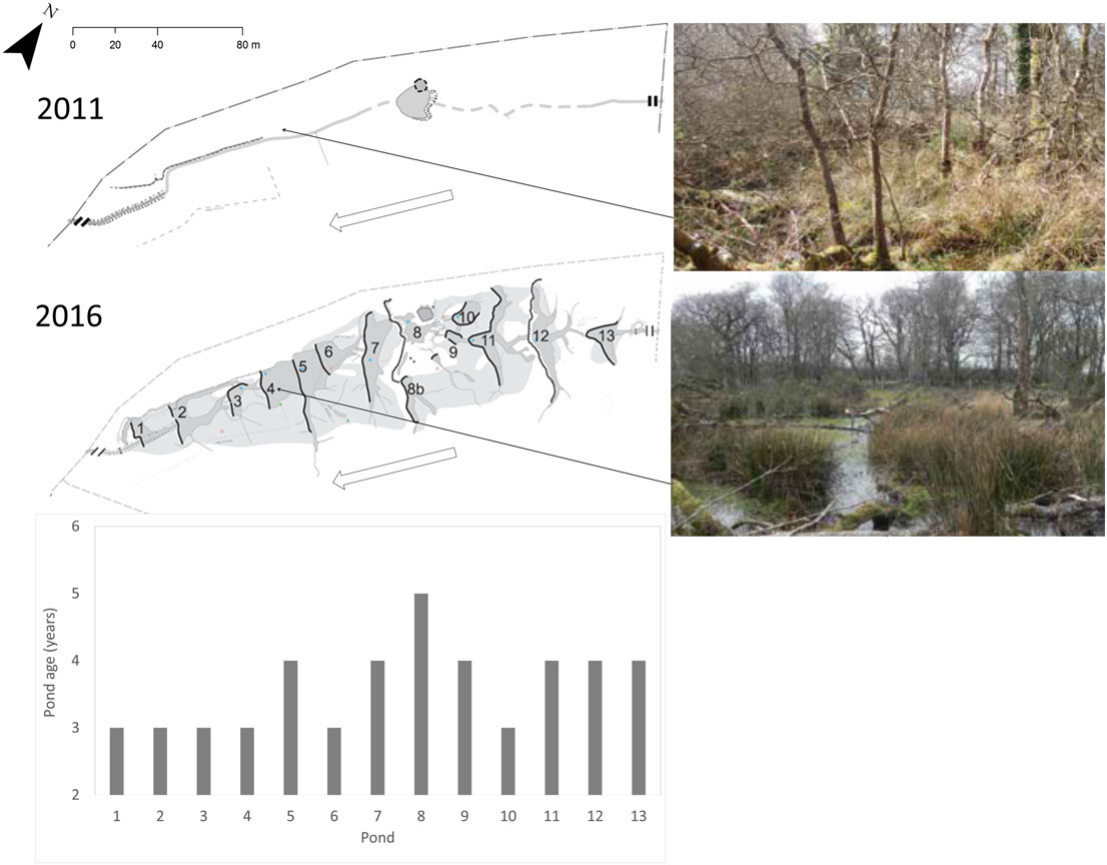
Schematic showing change in site structure between 2011 (immediately prior to beaver introduction) and 2016. Solid black lines signify dam position and extent while dark grey areas are impounded water and light grey areas are wet areas resulting from raised water table. Pond 8 was artificially constructed to allow for humane beaver release. Black and grey arrow indicates downstream flow direction through the site. Bottom graph illustrates age of ponds in years. Site schematics provided by South West Archaeology and included with permission. [Colour figure can be viewed at http://wileyonlinelibrary.com]

Surveying and sampling was undertaken at the Mid‐Devon Beaver Project controlled reintroduction site in Devon, South West England (DWT, 2013). The site is situated on a first‐order stream in the headwaters of the River Tamar catchment. The site has a 20 ha upstream catchment area dominated by intensively managed grassland. Drainage ditches around the perimeter hydrologically isolate the site, ensuring that the stream is the only flow in and out of the site and the only fluvial source of sediment and nutrients. Since beaver introduction, the site has changed from *c* 75% woodland cover (*Salix cinerea – Galium palustre* woodland) to a fen‐meadow dominated community (*Molinia caerulea – Cirisium dissectrum* fen meadow) (DWT, 2013). The site experiences a temperate climate with a mean annual temperature of 14°C and mean annual rainfall of 918 mm (Met Office, 2015). A pair of Eurasian beavers was introduced to the 1.8 ha enclosure, which includes a 183 m stretch of channel in 2011. As illustrated in Figure 1, prior to beaver reintroduction there were no ponds apart from pond 8, which was created to allow beaver reintroduction to the site. In the presented figures this constructed pond is displayed as Pond 8a and has since expanded to cover the area labelled 8b, which are analysed herein together as pond 8. Beaver activity has created a complex wetland environment, dominated by ponds, dams and an extensive canal network (DWT, 2013; Puttock *et al*., 2015). The age of ponds is detailed in Table 1.

**Table 1:** Summary pond characteristics alongside; mean, sum and normalised by area values of sediment (S) total nitrogen (N), total carbon (C), bulk density (BD). All errors are standard deviation (± SD). Pond positions are illustrated relative to data in Figures 2(a) and 3(a)

Pond and age (years)	Area 2016 (m^2^)	S Depth (m)	Volume S (m^3^)	C (%)	N %	BD (g cm^‐3^)	Sediment (t)	Carbon (t)	Nitrogen (t)
Pond 1 (3)	47.23	0.19±0.26	9.01±12.14	8.88±2.28	0.56±0.16	0.23±0.01	2.08±2.80	0.18±0.25	0.01±0.02
Pond 2 (3)	181.65	0.17±0.15	30.68±26.7	16.00±2.12	0.92±0.12	0.23±0.01	7.06±2.90	1.13±0.3	0.06±0.02
Pond 3 (3)	158.91	0.13±0.08	20.18±12.49	15.06±2.03	0.97±0.24	0.25±0.01	5.01±3.82	0.75±0.54	0.05±0.03
Pond 4 (3)	150.71	0.14±0.12	20.78±17.94	17.66±2.07	1.06±0.09	0.25±0.01	5.16±5.33	0.91±0.84	0.05±0.05
Pond 5 (4)	169.47	0.20±0.20	33.80±33.76	14.72±3.74	0.96±0.22	0.26±0.02	8.84±5.28	1.30±0.83	0.08±0.05
Pond 6 (3)	119.52	0.23±0.20	27.29±23.79	18.06±0.94	1.01±0.02	0.25±0.01	6.81±5.17	1.23±0.82	0.07±0.05
Pond 7 (4)	198.26	0.27±0.20	52.85±40.41	20.85±6.92	1.06±0.39	0.27±0.01	14.20±5.06	2.96±0.8	0.15±0.05
Pond 8 (5)	116.17	0.42±0.26	49.06±30.13	11.84±3.24	0.62±0.18	0.27±0.02	13.39±4.94	1.59±0.78	0.08±0.04
Pond 9 (4)	30.42	0.51±0.21	15.62±6.24	14.88±0.59	0.96±0.02	0.24±0.01	3.67±4.73	0.55±0.75	0.04±0.04
Pond 10 (3)	40.12	0.56±0.34	22.6±13.53	16.00±1.25	1.03±0.03	0.28±0.01	6.34±4.52	1.01±0.72	0.07±0.04
Pond 11 (4)	207.72	0.29±0.23	59.51±48.66	17.85±2.39	0.95±0.05	0.29±0.01	17.32±4.39	3.09±0.69	0.16±0.04
Pond 12 (4)	110.76	0.30±0.19	33.15±20.92	10.41±2.10	0.68±0.05	0.29±0.01	9.51±4.39	0.99±0.68	0.06±0.04
Pond 13 (4)	60.50	0.12±0.10	7.33±6.31	9.41±0.92	0.57±0.03	0.29±0.01	2.15±4.30	0.20±0.69	0.01±0.04
Mean (±SD)	122.42±61.77	0.27±0.15	29.37±16.28	14.74±3.65	0.87±0.19	0.26±0.02	7.81±4.72	1.22±0.9	0.07±0.05
Sum (±SD)	1591.44		381.87±93.02				101.53±16.24	15.90±2.50	0.91±0.15
Sum Normalised by Area (m^2^)			0.24				0.06	0.01	0.00

### Site survey and sample collection

As the site is constantly changing due to beaver activity, in addition to the long‐term monitoring of structural change delivered by annual surveys (shown in Figure 1), a survey was undertaken at the time of sediment sampling (October, 2016) to create a detailed ‘snapshot’ of the site structure. Pond extents were surveyed using a differential global positioning system (DGPS ‐ Leica GS08plus system). Sediment and water volumes within each pond were calculated via sampling at each node on a 2×2 m grid using a ranging pole (marked with mm increments). At each survey point the pole was gently inserted until the tip reached the top of the sediment layer, which was recorded as water depth. The pole was then gently pushed through the unconsolidated sediment until it reached a compacted layer, which was recorded as sediment depth as per Butler and Malanson (1995), Stefan and Klein (2004) and de Visscher *et al*. (2014). This method assumes the unconsolidated sediment layer to be material that has accumulated post‐pond creation while the compacted/consolidated layer is the pre‐pond surface. Surveying on a 2 m grid (at each node) resulted in a minimum of *n* = 12 (maximum *n* = 29) points being collected per pond.

At three randomly selected points within each of the 13 ponds, a core was taken through the sediment layer, using a beeker corer (Uwitec, Austria). Sediment was deposited into plastic bags and transported back to the University of Exeter's laboratories for analysis. In addition, the volume of samples was recorded allowing calculation of bulk density. For all variables, mean values for each pond were calculated using the three samples and are presented alongside standard deviation (Table 1). For total values (i.e. total sediment mass), a square root of the sum of squared SD values for each pond was used (i.e. total SD^2^ = (pond 1 SD^2^ + pond 2 SD^2^ +…)) to present a compiled SD value.

### Laboratory analysis

Upon collection, samples were oven dried (1 week at 40°C). The sample from a known volume was then dry weighed to calculate bulk density: (BD (g cm^3^) = dry sediment weight (g)/sediment volume (cm^3^). Samples were then sieved (<2 mm) and finely ground. Samples were analysed for carbon and nitrogen via dynamic flash combustion using a Flash 2000 Series and compared with standards of known value.

### Data processing and statistical analysis

To address Hypothesis 1 (Sediment and nutrient storage), sediment and nutrient volumes and mass within ponds, as well as the entire pond system, were calculated. As in Stefan and Klein (2004) and Butler and Malanson (1995), mean depths per pond (m) were combined with surveyed spatial extent (m^2^) allowing calculation of sediment and water volume at time of sampling. Mass of sediment was calculated by multiplying volume of sediment by bulk density and converted into tonnes (t):

(1) $$\mathrm{Sm}=\left(V\times \mathrm{BD}\right)$$

where *Sm* = sediment mass (g), *V* = volume (m^3^), *BD* = bulk density (g m^3^).

Further analysis was undertaken to understand storage of sediment within the site. As in previous studies (Butler and Malanson, 1995; Stefan and Klein, 2004), annual accumulation rates were calculated by dividing average sediment depth (m) by age (years). Normalised by area (m^2^) values were calculated by dividing volume and mass calculations by surface area of each pond. The total pond volume at time of sampling was calculated as the sum of water and sediment volumes to understand the remaining potential storage capacity of ponds at time of sampling.

Percentage carbon and nitrogen values for each pond were used to calculate carbon‐to‐nitrogen ratios (C:N) and also total mass of carbon and nitrogen stored within each pond. As in previous studies (Peukert *et al*., 2012; Glendell *et al*., 2014), nutrient stocks (carbon and nitrogen) were calculated by multiplying mean pond decimal percentage concentrations (%, *n* = 3), with bulk density (g m^3^) and volume (m^3^) and then converting to tonnes:

(2) $$\mathrm{Ns}=\left(V\times \mathrm{BD}\times \left(n÷100\right)\right)$$

where *Ns* = nutrient stock (carbon or nitrogen (g), *V* = volume (m^3^), *BD* = bulk density (g m^3^) and *n* = nutrient percentage concentration (carbon or nitrogen).

To address Hypothesis 2 (Storage downstream) and Hypothesis 3 (Storage and age), statistical analysis was undertaken between ponds (*n* = 13). Exploratory analysis illustrated that data were not normally distributed and were therefore log transformed for normality. To establish whether observed variance between ponds was statistically significant, an independent two‐tailed heteroscedastic *t*‐test was used. The tests assumed unequal variance between samples and was carried out at the 95% confidence level (*P <* 0.05). Relationships between measured pond variables were tested using linear regression while correlations between downstream pond position and measured variables were undertaken on non‐normalised data using the non‐parametric Spearman's rank correlation. All tests were undertaken using SPSS v23 (SPSS Inc, IBM, USA). Unless otherwise stated, all errors are standard deviations around the mean (detailed for measured variables in Table 1 and Table 2).

**Table 2:** An illustration of total pond volume and remaining storage capacity at a point in time (October 2016) if the system was to remain static. All errors are standard deviation (±SD). Pond positions are illustrated relative to data in Figures 2(a) and 3(a)

Pond and age (years)	Volume Water (m^3^)	Volume Sediment (m^3^)	Total Pond Volume (m^3^)	% Remaining Capacity Volume	Extra sediment capacity (t)
Pond 1 (3)	16.45±6.51	9.01±12.14	25.46±11.45	64.61±23.04	3.8±1.5
Pond 2 (3)	77.81±31.5	30.68±26.7	108.49±32.05	71.72±20.21	17.91±7.25
Pond 3 (3)	44.49±17.41	20.18±12.49	64.68±21.74	68.8±12.7	11.05±4.32
Pond 4 (3)	60.68±29.56	20.78±17.94	81.46±24.44	74.49±21.09	15.06±7.34
Pond 5 (4)	43.12±32.79	33.8±33.76	76.92±42.21	56.06±27.42	11.28±8.57
Pond 6 (3)	34.2±25.75	27.29±23.79	61.49±31.71	55.62±21.95	8.53±6.42
Pond 7 (4)	43.62±24.18	52.85±40.41	96.46±45.29	45.22±24.36	11.72±6.49
Pond 8 (5)	27.06±11.2	49.06±30.13	76.13±31.48	35.55±21.11	7.39±3.06
Pond 9 (4)	10.65±4.48	15.62±6.24	26.26±10.29	40.54±5.35	2.5±1.05
Pond 10 (3)	14.51±0.86	22.6±13.53	37.11±13.51	39.1±16.24	4.07±0.24
Pond 11 (4)	62.32±33.3	59.51±48.66	121.83±59.7	51.15±23.63	18.13±9.65
Pond 12 (4)	29.02±15.62	33.15±20.92	62.17±23.47	46.67±26.63	8.32±4.48
Pond 13 (4)	15.8±5.36	7.33±6.31	23.12±7.72	68.31±15.73	4.63±1.57
Mean (±SD)	36.90±20.09	29.37±15.64	66.28±30.69	55.22±12.83	9.58±5.02
Sum (±SD)	479.72±77.86	381.87±93.02	861.58±111.90		124.39±2.03

It has been shown that there will be some sediment sourced from beaver building activity and within site erosion (Lamsodis and Ulevičius, 2012; de Visscher *et al*., 2014; Hood and Larson, 2014). Sediment partitioning or source determination was not undertaken as part of this study. Over such small contributing areas (20 ha headwater catchment in this case) there is very little discriminatory power in existing techniques and considerable uncertainty associated with estimates of sediment source (Smith and Blake, 2014). Instead, to address the source of sediment in ponds (Hypothesis 4), data describing sediment mass in ponds recorded in this study, were combined with hydrological and water quality data previously published from the site (Puttock *et al*., 2017) to estimate upstream catchment contributions to the quantities of sediment and nutrients stored in the beaver ponds.

In previous work undertaken at the study site (see Puttock *et al*., 2017, for full details), 226 water quality samples were collected between 2014 and 2015. These samples were collected through a full range of flow conditions (from baseflow to peak flow) across 11 storm events both for water entering the site ‘Above Beaver’ and water leaving the site after travelling through the pond complex ‘Below Beaver’. This sampling, while giving an insight into the differences in water quality entering and leaving the site, did not give enough temporal coverage to calculate total sediment loadings for the duration of the 5 years since beaver introduction, for example, using Walling and Webb (1985) method. Therefore, the difference between mean suspended sediment values Above Beaver (112.42 ± 71.47 mg L^−1^) and Below Beaver (39.15 ± 36.88 mg L^−1^), combined with annual discharge entering the site over the monitoring period (2014–2015) was used to approximate sediment yield from the upstream catchment (Equation (3)) and furthermore, calculate an estimated annual erosion rate (Equation (4)).

(3) $$\mathrm{SC}=\left(\frac{\mathrm{SS}\times Q}{1e^{+09}}\right)\times T$$

where *SC* = sediment from catchment (t); *SS* = difference in suspended sediment Above Beaver and Below Beaver (mg L^‐1^) *Q* = discharge for a 1 year period (L) and *T* = time beavers have been at the site (years).

(4) $$\mathrm{AR}=\left(\frac{\mathrm{SC}}{C}\right)/T$$

where *SC* = sediment from catchment (t); *AR* = mean annual erosion rate (t ha^‐1^ yr^‐1^); *C* = catchment size (ha) and *T* = time beavers have been at site (years).

## Results

### Total sediment and nutrient storage

Ponds covered a total of 1591 ± 61.77 m^2^ of the 1.8 ha study site (i.e. surface water covered 9% of the land area). The 13 ponds had a mean total depth of 0.58 ± 0.16 m, a mean water depth of 0.31 ± 0.07 m and a mean sediment depth of 0.27 ± 0.15 m. Given the site had been active for 5 years at the time of sampling (although there is some variation in pond age from 3 to 5 years), this equates to an average annual accumulation rate of 5.4 ± 3.0 cm yr^‐1^. In total, the ponds stored 381.87 ± 16.28 m^3^ of sediment which, when combined with bulk density values (mean 0.26 ± 0.02 g cm^3^) equated to a total of 101.53 ± 16.24 t of sediment within the 13 ponds. As shown in Figure 1, prior to beaver reintroduction, there were no ponds at the site and even if Pond 8, which was artificially created to facilitate beaver introduction to the site is not included, this represents a sediment storage increase of 88.14 t in 5 years. Normalised per ponded area, the site stores an average of 71.40 ± 39.65 kg of sediment per m^2^ of pond. The ratio of remaining storage capacity to measured water level was also calculated, with the assumption that the site was to remain static with no further beaver engineering. Results presented in Table 2 indicate that, overall the pond system had a remaining 55.7% potential storage capacity, equating to 124.4 t of sediment.

Analysis of this sediment showed mean percentage concentrations of 14.74 ± 3.65% total carbon and 0.87 ± 0.19% total nitrogen, equating to total storage of 15.90 ± 2.50 t of carbon and 0.91 ± 0.15 t of nitrogen within the ponds.

### Changes in sediment and nutrient storage through the pond sequence

It was hypothesised that, in a sequence of ponds, sediment and nutrient storage would change downstream (Hypothesis 2). Variability, between ponds and downstream through the pond sequence, was investigated. Table 1 summarises survey results quantifying the surface area of ponds, in addition to the quantity of sediment and water being stored at the time of fieldwork.

**Figure 2:**
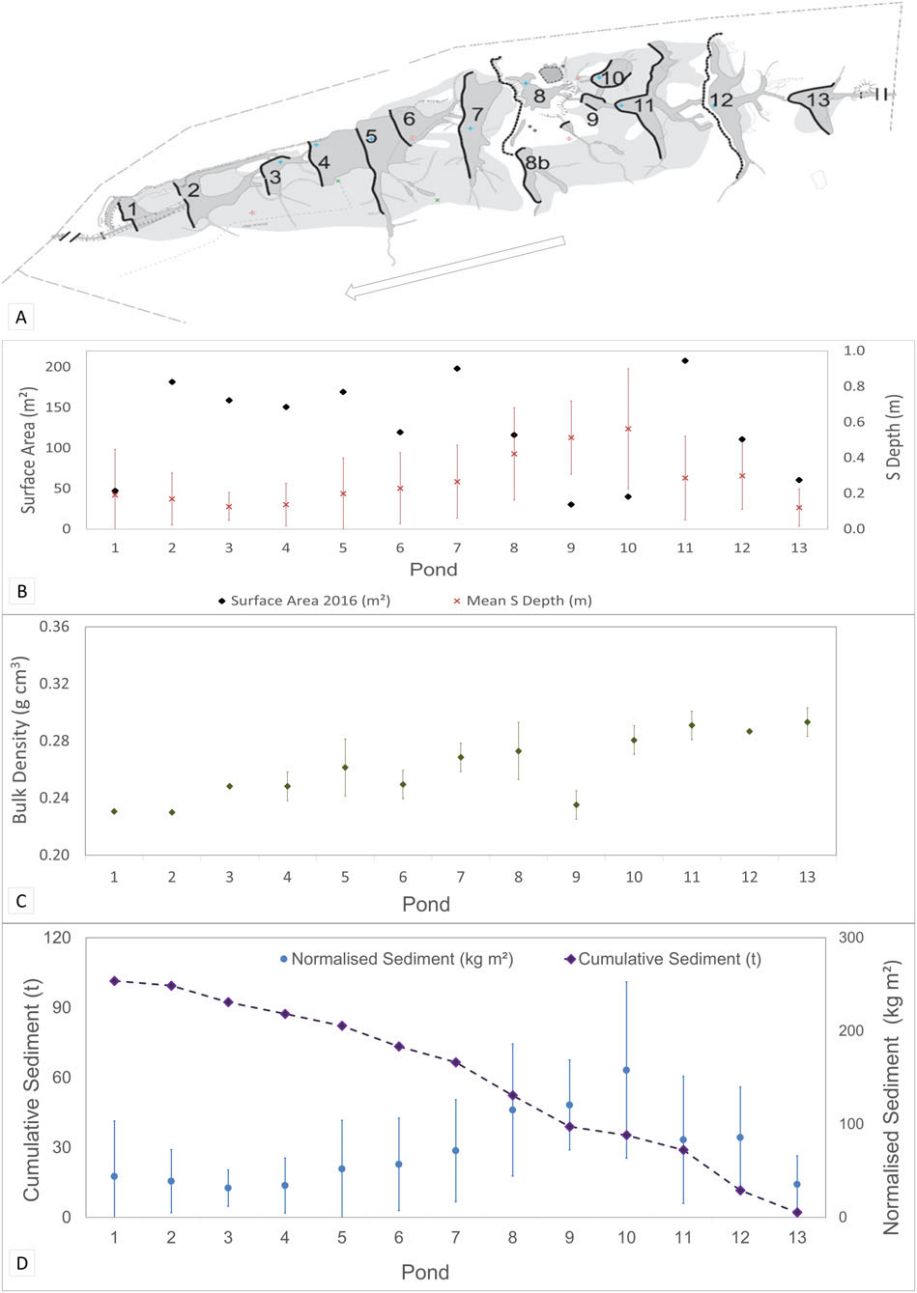
Pond sediment survey results. (a) 2016 pond schematic with ponds numbered and arrow indicating flow direction. Provided by South West Archaeology and included with permission; (b) pond characteristics including sediment depth and surface area; (c) bulk density throughout the pond sequence; and (d) cumulative sediment throughout the sequence and normalised sediment per m^2^ surface area. [Colour figure can be viewed at http://wileyonlinelibrary.com]

Figure 2(B) illustrates how factors contributing to total sediment and nutrient storage (pond area, sediment depth and bulk density) change downstream throughout the sequence of 13 ponds. Neither surface area nor depth showed a significant relationship with downstream position (*P* > 0.05). In contrast, bulk density showed an overall marginal, but statistically significant downstream increase (*P* < 0.05, r^2^ = 0.67). The amount of sediment in individual ponds related closely to the surface area of ponds with bigger ponds storing more sediment (*P* < 0.05, r^2^ = 0.45), regardless of location within the site.

To explore further how sediment storage varies with distance downstream, normalised sediment storage values per ponded surface area (m^3^ per m^2^ and kg per m^2^) were calculated. Overall, there was no significant correlation between normalised pond sediment values and downstream position (*P* > 0.05). However, as can be seen from Figure 2(C) normalised sediment per m^2^ and sediment depth showed a notable spike being significantly higher (*P* < 0.05) between ponds 12 and 7, compared with the first pond (13) and downstream ponds (6–1). The downstream ponds also showed a significantly higher (*P* < 0.05) mean remaining storage capacity (65.2%) than the site as a whole (55.7%).

As outlined in Hypothesis 3, it was hypothesised that the age of each pond could impact upon sediment storage, with older ponds having had more time to accumulate sediment. The age of ponds (Figure 1) was determined from previous surveys undertaken at the site. The ponds that had been present longest (4–5 years), showed significantly higher total amounts of sediment (*P* < 0.05) and higher (but not significantly, *P* > 0.05) normalised sediment values than newer ponds (≤3 years).

**Figure 3:**
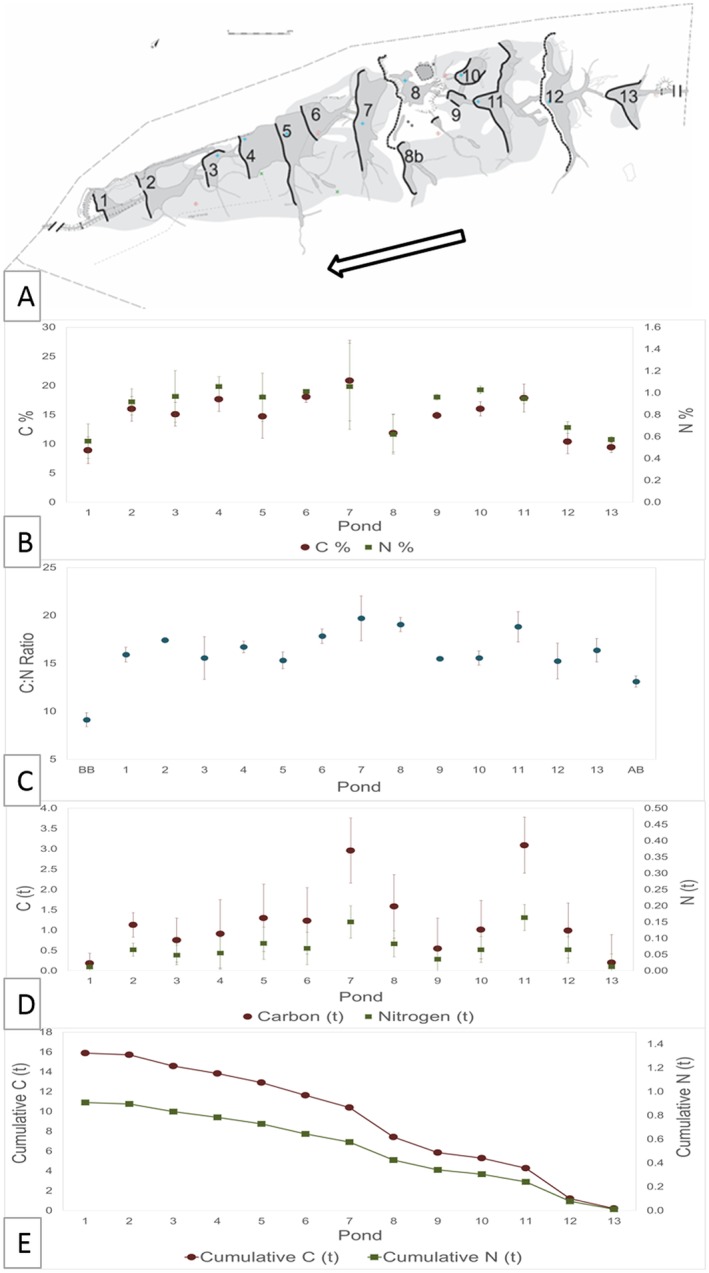
Pond sediment carbon and nitrogen content results. Top (a) 2016 pond schematic with ponds numbered and arrow indicating flow direction. Provided by South West Archaeology and included with permission; middle top (b) C:N ratios throughout the pond sequence and above the site (AB) and below the site (BB); (c) carbon and nitrogen concentrations throughout the pond sequence and above the site (AB) and below the site (BB); middle bottom (d) total carbon and nitrogen within each pond; bottom (e) cumulative total carbon and nitrogen throughout the pond sequence. [Colour figure can be viewed at http://wileyonlinelibrary.com]

Nutrient stores associated with sediment also varied significantly across the study site. As illustrated in Figure 3(B), mean percentage concentrations of both carbon and nitrogen in pond sediment (C = 14.74 ± 2.35; *N* = 0.87 ± 0.12, *n* = 39) were significantly higher (*P* < 0.05) than mean percentage concentrations of channel bed sediment, both upstream and downstream of the beaver‐impacted site (C = 1.56 ± 0.20%; *N* = 0.13 ± 0.02%, *n* = 6). In addition, both carbon and nitrogen showed higher percentage concentrations in sediment entering the site Above Beaver (AB; C = 2.40 ± 0.33%; *N* = 0.18 ± 0.03%, n = 3), compared with Below Beaver (BB; C = 0.72 ± 0.06%; *N* = 0.08 ± 0.003%, n = 3).

Significant differences in mean percentage concentrations of carbon and nitrogen were observed between ponds (*P* < 0.05). However, for both nutrients, there was no significant correlation with downstream position or volume/mass of sediment in ponds (*P* > 0.05). Total mass of carbon and nitrogen in ponds (Figure 3(D)) showed a significant positive correlation (*P* < 0.05) with pond surface area and also volume/mass of sediment (*P* < 0.05) although the latter cannot be considered as an independent variable.

For both concentrations and total mass, carbon and nitrogen showed a strong positive relationship with each other (*P* < 0.001). C:N ratios showed no significant difference throughout the pond sequence (*P* > 0.05). However, within pond C:N ratios were slightly higher within pond sediment than sediment above (*P* > 0.05) and significantly higher than sediment below the pond sequence (*P* < 0.05).

### Source of sediment in beaver ponds

If the beaver ponds had a trapping efficiency of 100% and 100% of sediment trapped in the beaver ponds (101.53 t) was sourced from the upstream catchment this would equate to 20.3 t yr^‐1^ being lost from the 20 ha catchment, over the 5 year period since beaver introduction (or an erosion rate of 0.98 t ha yr^‐1^). However, zit was hypothesised that sediment and nutrients stored in ponds is sourced from both in‐site redistribution and sediment eroded from intensively managed grassland upstream, but is dominated by the latter (Hypothesis 4).

In previous research at the site, mean suspended sediment values of 112.42 ± 71.47 mg L^−1^ were reported in water entering the site and a mean of 39.15 ± 36.88 mg L^−1^ in water leaving the site (Puttock *et al*., 2017). These results suggest a net trapping efficiency (or overall downstream reduction in suspended sediment concentrations) of 65.17%.

Applying Equation (3), given a difference in suspended sediment of 73.35 mg L^‐1^ and a total annual discharge of 1.95E+08 L (Puttock *et al*., 2017) for the monitoring period, equates to an estimated 71.42 t or 70.34% of the total sediment in ponds being sourced from the catchment upstream. Applying Equation (4) to this 71.42 t of sediment, estimated to have originated from the upstream catchment (of 20 ha), results in an estimated annual rate of 0.71 t ha^‐1^ yr^‐1^ over a 5 year period.

## Discussion

### Total sediment and nutrient storage

It is clear that beaver activity at the study site has resulted in dramatic structural change and significant amounts of both sediment and nutrients being stored within the 13 ponds. The total of over 100 t of sediment combined with almost 16 t of carbon and 1 t of nitrogen supports Hypothesis 1 that beaver ponds act as large sediment and nutrient stores. This supports previous research finding that beaver impoundments create localised sediment deposits, having the ability to accumulate large volumes of sediment and associated nutrients (Butler and Malanson, 2005; Law *et al*., 2016). It is further evident that beaver ponds change not just the hydrological regime of small channels, by slowing flow and enhancing water storage (Puttock *et al*., 2017), but also create landscapes with depositional sediment regimes (Burchsted *et al*., 2010), as signified by the large sediment volumes recorded in this study.

Data herein illustrates nutrient storage associated with beaver pond development, both in terms of carbon and nitrogen deposition. These results support the existing body of research showing that wetlands, in the broad sense, act as valuable sediment and nutrient stores (Johnston, 1991), particularly in contrast to anthropogenic degraded landscapes (Nahlik and Fennessy, 2016). Furthermore, results indicate that beaver engineered wetlands are exemplars of such valuable wetlands and can successfully exist or be created within intensively managed European agricultural landscapes (Law *et al*., 2017; Puttock *et al*., 2017).

The large mass of sediment (101.53 ± 4.72 t or 71.40 ± 39.65 kg per m^2^ of ponded extent) being stored in a relatively small area (1.8 ha) in this study is in agreement with previous studies, primarily from North America. Low order streams, containing dams have previously been shown to account for up to 87% of sediment storage at reach scales (Hering *et al*., 2001), while the removal of a sequence of beaver dams in Sandon Creek, British Colombia, led to the mobilisation of 648 m^3^ of stored sediment (Butler and Malanson, 1995, 2005). Butler and Malanson *et al*. (1995) also reported a range of 2–28 cm yr^‐1^ of sediment accumulated in several beaver ponds in Glacier National Park, Montana, while for six different ponds (also in Glacier National Park, similar rates of *c*. 4–39 cm yr^‐1^ were reported (Butler and Malanson, 1994). Values of sediment accumulation from North American beaver systems indicate the estimated average accumulation value of 5.4 cm yr^‐1^ presented in this study may be at the lower end of what is possible in bigger dam–pond complexes or systems with a more plentiful sediment supply. In one of the few studies in European landscapes, De Visscher *et al*. (2014), studied sediment accumulation in two beaver pond sequences in predominately extensively‐managed forest/meadow ecosystems of the Chevral River, Belgium. de Visscher *et al*. (2014) estimated the total sediment mass deposited in the dam sequences at 495.9 t. From the two pond sequences, average pond area was 200.4 m^2^, average sediment depth 25.1 cm and average sediment mass of 14.6 t, equating to a normalised mass of 72.65 kg of sediment m^2^. These values are very similar to the mean sediment depth of 27 cm and mean normalised mass of 71.40 kg m^2^ reported in this study from the UK, albeit from entirely different ecosystems. The sediment accumulation values presented both in this study and others, also demonstrate that beaver ponds can exhibit high sediment accumulation values in comparison with other wetland systems. As an example, in a review of sediment accumulation rates in freshwater wetlands (Johnston, 1991) a mean annual accumulation rate of 0.69 cm yr^‐1^ was reported across 37 different wetland types, ranging from riparian forest to wet meadows.

As long as supply continues, sediment will continue to accumulate until either the pond infills and sediments are colonised by plants forming a beaver meadow (Polvi and Wohl, 2012) or a dam collapses releasing sediment (Butler and Malanson, 2005). In catchments with high stream power, and associated risk of dam failure, there may be lower and less stable long‐term sediment associated stores of nutrients than presented herein (Błȩdzki *et al*., 2011). However, where local factors, such as channel gradient, support the stable construction of dams and the resulting stream discontinuity, nutrients may be retained in sediments as shown in this study. Plant colonization and the creation of beaver meadows can further immobilise these sediments and associated nutrients (Naiman *et al*., 1994). Furthermore, as a considerable volume of potential storage capacity within the 13 yet remains (> 55%), without accounting for ongoing dam building, it may be expected that beaver damming continues to enhance or at least maintain a dynamic equilibrium of sediment storage at the site (Giriat *et al*., 2016).

It is notable that, at the site reported here, dam failures and resulting sediment releases have not been observed since beaver release. However, dam failures, particularly in high energy environments, may cause infrequent but significant pulses of sediment (Butler and Malanson, 2005). Such pulses may, in some cases, exert significant impacts upon river geomorphology (Bigler *et al*., 2001; Butler and Malanson, 2005). However, different sediment retention dynamics have been reported following dam collapse. Giriat *et al*. (2016) found that there were very minimal losses of sediment from the Beaver ponds studied, following a dam collapse. Similarly, Butler and Malanson (2005) reported that the majority of sediments were retained in ponds and subsequently stabilised following colonisation and dam reconstruction. Levine and Meyer (2014) reported large sediment losses but the remnants of the dam structure were found to trap sediment, which was rapidly colonised by plants and stabilised. In contrast, other studies have observed rapid loss of pond sediments following dam collapse (Curran and Cannatelli, 2014; Levine and Meyer, 2014). It is likely that, as with the site studied, where closely‐spaced, multi‐dam complexes exist, these will provide a major buffering effect, reducing the likelihood of dam failure and, in so doing, also reducing the downstream release of sediment from any single dam failure. It is clear from the literature that significant uncertainty regarding dam failure dynamics exists (Anderson and Shaforth, 2010; Klimenko and Eponchintseva, 2015) and is an area in need of further research.

Research undertaken in this study suggests that sediment is enriched in both carbon and nitrogen (average across all ponds of 14.74% C and 0.87% TN), resulting in a notable store of nutrients within the landscape. This summary is supported by previous research and is commonly attributed to the same factors such as channel discontinuity and flow velocity reduction that result in sediment deposition and storage of associated nutrients (Naiman *et al*., 1986; Devito *et al*., 1989; Lizarralde *et al*., 1996; Klotz, 2013). Wohl (2013) estimated that even relict beaver dam‐related storage can account for 8% of total carbon storage within the landscape and actively maintained beaver wetlands up to 23%.

Compared with semi‐natural ecosystems, intensive agricultural landscapes are often depleted in carbon (Webb *et al*., 2001; Quinton *et al*., 2006). The proportions of nutrients in sediment entering the site (carbon 2.4 ± 0.3% nitrogen 0.18 ± 0.03%) are lower, but comparable with those reported in Peukert *et al*. (2016) for three intensively managed grassland field systems on similar soil types and in comparable topographic locations, in the South West UK (total carbon range: 3.5–5.0% and total nitrogen range 0.4–0.6%). Such findings, in addition to high within‐site storage values, suggest that even when agricultural source areas are depleted in carbon, beaver ponds can still play a role in enhancing carbon storage in the landscape. Therefore, beaver dams may recreate valley bottom wetlands, which would have historically been nutrient rich (Wohl, 2013).

There is only a limited amount of research into the nutrient storage associated with sediment stored in beaver ponds and even less from intensively‐managed agricultural landscapes. A key area that is unclear and beyond the scope of this study, is how the impoundment of water, sediments and associated nutrients in ponds affects biogeochemical cycling and the resulting transfers of nutrients in both gaseous and dissolved forms. Previous research at the study site (Puttock *et al*., 2017) showed that compared with water entering the site, water leaving the site had lower levels of both suspended sediment and also nitrogen. Naiman *et al*. (1994) found that following the build‐up of large nitrogen stocks in ponds, there is some removal through both transport and local cycling; however, the majority of nitrogen is retained in pond sediments and taken up by plants. Similarly Correll *et al*. (2000) showed that, before dam construction, nitrogen concentrations were significantly correlated with river discharge but, after dam construction, no significant relationship was observed; perhaps due to enhanced plant uptake or degassing of CH_4_ and N_2_O.

In contrast to nitrogen values, dissolved organic carbon levels have been shown to be higher leaving the site than entering (Puttock *et al*., 2017). This was attributed to the greater carbon stocks within site in contrast to the relatively carbon depleted soils in the agricultural catchment upstream. This finding is supported by previous work showing beaver ponds retain organic matter (Law *et al*., 2016) and consequently act as net carbon stores (Lizarralde *et al*., 1996; Correll *et al*., 2000), but attributing increased dissolved organic carbon (DOC) downstream of beaver ponds to increased primary production in ponds (Correll *et al*., 2000). Beaver ponds have also been shown to result in increased carbon dioxide and methane fluxes compared with non‐impacted river reaches (Vecherskiy *et al*., 2011; Lazar *et al*., 2015), although It has been suggested that the sequestration of carbon‐rich sediment in ponds may help offset any increase in gaseous carbon emissions associated with ponds (Johnston, 2014). From previous studies there is some inconsistency in the reporting of retention, production and release of both carbon and nitrogen in beaver ponds with climatic and seasonal variation in temperature and discharge, pond age and level of plant colonisation likely to be key controls (Devito *et al*., 1989; Naiman *et al*., 1994).

### Changing sediment and nutrient storage through the pond sequence

Beaver pond sequences are heterogeneous and the number, characteristics and distribution of ponds may have significant implications for sediment and nutrient storage. The distribution and properties of sediments within ponds and along pond complexes is discussed by several authors (Gurnell, 1998; Walsh *et al*., 1998; Meentemeyer and Butler, 1999; Bigler *et al*., 2001; de Visscher *et al*., 2014), though there is notable variability between studies. Beaver pond size will depend on the characteristics of the catchment, building material available, as well as the size of stream in which they occur (Butler and Malanson, 1995; de Visscher *et al*., 2014). Previous research has determined that pond infilling can also be a function of dam age (Meentemeyer and Butler, 1999; Bigler *et al*., 2001), with older ponds typically accumulating more sediment (Gurnell, 1998). Herein, the older ponds appeared to hold more sediment, supporting Hypothesis 3 that storage is positively correlated with age, but this relationship was non‐significant. This is probably due to the relatively low number of ponds and low difference between maximum ages with ponds at similar successional stages (Naiman *et al*., 1988).

A common finding in previous studies is that larger ponds (by surface area) hold more sediment (Butler and Malanson, 1995; Walsh *et al*., 1998; Giriat *et al*., 2016). Herein, no matter where the ponds are located behind the sequence of 13 dams, larger ponds not only hold significantly more total sediment, but also hold more sediment per unit area. These results suggest that larger ponds may exert a greater influence on flow dynamics and sedimentation patterns, with de Visscher *et al*. (2014) explaining this via velocity gradients across ponds.

In addition to size, the position of each pond within a series of ponds may play a role in sediment and nutrient storage (Hypothesis 2). Studies have identified that there is a downstream decrease in storage between ponds, with the most upstream ponds storing more than those downstream (Butler and Malanson, 1995; Stefan and Klein, 2004). This has been attributed to high energy upstream catchments providing a sediment supply which accumulated more rapidly in the upstream ponds. In a lower energy environment, no difference in sedimentation might be observed between ponds because the majority of sediment would be fine and transported in suspension; therefore, larger ponds were found to retain the largest volumes (Butler and Malanson, 1995). Being in a first order, headwater tributary, it may be anticipated that the study site examined herein falls into the latter category, as supported by the relationship between sediment and pond size. However, as illustrated in Figure 2, sediment mass normalised by area shows a distinctive pattern with a peak in the middle ponds. Water entering the site during storm events (when sediment loads are highest) may have the energy to carry sediment through the first pond, before it is slowed in subsequent ponds depositing sediment. Water entering the downstream ponds is sediment depleted resulting in less sediment being deposited in the lower ponds and lower concentrations of suspended sediment leaving the site (Puttock *et al*., 2017). Therefore, results suggest that, in addressing Hypothesis 2, downstream position does play a role in sediment storage.

Bulk density values reported in previous research range from 0.47 ± 0.05 g cm^3^ by Naiman *et al*. (1994) to 0.29 ± 0.05 g cm^3^ by de Visscher *et al*. (2014), with the mean values reported in this study (0.26 ± 0.02 g cm^3^), being marginally lower than this range. Previous studies including that by Naiman *et al*. 1994), also recorded no significant change in bulk density throughout the pond sequence. In this study a small, but statistically significant downstream increase in bulk density was observed, which combined with the previously discussed reduction in sediment depth in the lower ponds, adds to a picture of sediment being preferentially trapped and deposited in the upper to middle ponds (Butler and Malanson, 1995), with less sediment in lower ponds.

Total carbon and nitrogen at the study site varied with the size of pond and mass of sediment. Nutrient concentrations within sediment showed no discernible change throughout the pond sequence. Both carbon and nitrogen concentrations in ponds were significantly higher (*P* < 0.05) than samples taken from within channel locations above and below the beaver‐impacted site. Concentrations and C:N ratios in sediment above the pond sequence were higher than those leaving the site, indicating preferential in‐site carbon retention. Lizarralde *et al*. (1996) found that sediment trapped in beaver ponds contained a greater concentration of nutrients, including carbon, than riffle environments in the same reach. Similarly, Johnston (2014) found beaver ponds to exhibit higher nutrient concentrations than adjacent unimpounded soils.

### Sources of sediment in beaver ponds and wider implications

While the source of much of the beaver pond sediment appears to be the upstream catchment, beaver activity within the site has undoubtedly contributed. It has been shown that beaver activity can constitute a sediment source primarily through the contribution of excavated material from burrows and canals (Lamsodis and Ulevičius, 2012). Attempts have been made to quantify such sources; for example, Lamsodis and Ulevicius (2012) investigated the contribution of beaver (C. fiber) excavation to sedimentation in lowland agricultural ditches in Lithuania. They found that, in a given 1 km reach of beaver‐impacted channel, a mean of 53 burrows were observed which could generate an estimated 80 m^3^ of sediment (approximate volume of 1.49 m^3^ per burrow). Another study (focusing on C. canadensis), by Butler and Malanson (1995) suggests a lower, but still noteworthy value of 0.4 m^3^ per burrow (Butler and Malanson, 1995). Similarly, in a study of C. canadensis in 16 US wetlands, it was found that the contribution of sediment from beaver canals to rivers was significant (Hood and Larson, 2014). The authors show that, over a 13 km^2^ area in the Miquelon Lake Provincial Park, Canada, an estimated 22 315 m^3^ of sediment was released into the watercourse. Erosion from within ponds (de Visscher *et al*., 2014) or dam failure (Butler and Malanson, 2005) upstream in a dam sequence may also contribute sediment of a mixed source to ponds downstream.

It is probable that the ratio between beaver sourced sediment and other sources of sediment, such as anthropogenic soil erosion, will vary greatly as a function of land use, existing channel characteristics and beaver population densities. Similarly, the overall contribution of beaver activities to reach or catchment scale sediment budgets will vary greatly depending on the extent and nature of beaver engineering activities. It may be hypothesised that in reaches where extensive and stable dam structures exist, the ability of beaver activity to act as a sediment sink may be significant. In contrast, in areas where beavers exist but are not damming, their burrowing and other activities may act as a sediment source that is rarely quantified in existing monitoring and management strategies.

The results presented here support the acceptance of Hypothesis 4 (Sediment source), they show that over 70% (or *c*. 70 t) of the sediment stored in the ponds was sourced from the upstream intensively‐managed grassland catchment over the course of 5 years. The calculated annual rate of 0.71 t ha^‐1^ yr^‐1^ equates closely to that of 0.72 t ha^‐1^ yr^‐1^, which was reported as a mean annual erosion rate for intensively managed grasslands (from nine studies) in a recent compilation of UK soil erosion studies (Benaud *et al*., 2017).

Globally, soil erosion and degradation of predominately agricultural land is both an environmental and economic threat (Gregory *et al*., 2015). Erosion is also a serious issue for downstream water quality leading to siltation, habitat destruction and eutrophication (Bilotta *et al*., 2008). While beaver channel modification cannot prevent agricultural soil erosion, the reintroduction of beavers into headwaters may provide a means by which to trap sediment (and associated nutrients) in ponds and reconnect floodplains, limiting negative downstream impacts. For example, in North America beavers are increasingly used as a cost‐effective restoration tool to restore incised and eroding stream systems (Pollock *et al*., 2014) and also to restore channel heterogeneity and fish habitat (Bouwes *et al*., 2016). Results presented herein go some way to demonstrating that this could also be a viable strategy within the agricultural landscapes which prevail in Western Europe.

In the UK, the value of wetland recreation is recognised (Braskerud *et al*., 2005; Deasy *et al*., 2009), with recommendations for wetland creation across 2% of catchments having being made (Millhollon *et al*., 2009). Others have suggested smaller, strategically placed features could play a key role (Braskerud *et al*., 2005; Ockenden *et al*., 2014). However, such work commonly focuses on anthropogenic features with associated construction and maintenance costs (Ockenden *et al*., 2012). Allowing the recreation of more natural environments, may provide a cost‐effective strategy (i.e. when beavers constantly maintain active dam sequences to maintain water storage capacity), while additionally providing a host of other benefits such as biodiversity and habitat restoration (Law *et al*., 2017), flow attenuation and water quality improvements (Puttock *et al*., 2017). The estimated sediment accumulation rates, presented for the pond sequence in our study (0.71 t ha^‐1^ yr^‐1^), compares closely with those presented by Ockenden *et al*. (2012) for 10 different wetlands constructed with the aim of sediment retention (range 0.01–0.8 t ha^‐1^ yr^‐1^).

## Conclusion

Results presented in this paper illustrate that beavers can exert a significant impact upon sediment and nutrient storage. Beaver ponds were shown to hold large volumes of sediment and associated nutrients. Results also suggest that, whilst pond age and deposition in a dam–pond sequence may play a role in sediment and nutrient storage, the clearest control was pond size, with larger ponds holding more sediment per unit area.

Unlike most previous work, this study focused on a site located within an intensively managed grassland landscape. It was inferred that the majority of sediment trapped in the ponds originated from erosion in the upstream intensively managed grassland catchment, therefore, beaver dams mitigated the loss of this sediment downstream. While further understanding of the long‐term stability of sediment and nutrient storage in beaver ponds is now required, findings presented in this study have important implications for understanding the role beavers may play as part of catchment management strategies.

## References

[esp4398-bib-0001] Andersen DC , Shafroth PB . 2010 Beaver dams, hydrological thresholds, and controlled floods as a management tool in a desert riverine ecosystem, Bill Williams River, Arizona. Ecohydrology 3: 325–338. 10.1002/eco.113.

[esp4398-bib-0003] Benaud P , Anderson K , Carvalho J , Evans M , Glendell M , James M , Lark M , Quine, T , Quinton, J , Rawlins, B , Rickson J , Truckell I , Brazier R . 2017 What can we learn from national‐scale geodata describing soil erosion? 19th EGU General Assembly, EGU2017, proceedings from the conference held 23–28 April 2017 in Vienna, Austria, 9050 **19**: 19050. [online] Available from: http://adsabs.harvard.edu/abs/2017EGUGA..1919050B

[esp4398-bib-0004] Bigler W , Butler DR , Dixon RW . 2001 Beaver‐pond sequence morphology and sedimentation in Northwestern Montana. Physical Geography 22: 531–540. 10.1080/02723646.2001.10642758.

[esp4398-bib-0005] Bilotta GS , Brazier RE , Haygarth PM , Macleod CJ , Butler P , Granger S , Krueger T , Freer J , Quinton J . 2008 Rethinking the contribution of drained and undrained grasslands to sediment‐related water quality problems. Journal of Environmental Quality 37: 906–914. 10.2134/jeq2007.0457. 18453413

[esp4398-bib-0006] Bilotta GS , Krueger T , Brazier RE , Butler P , Freer J , Hawkins JMB , Haygarth PM , Macleod CJA , Quinton JN . 2010 Assessing catchment‐scale erosion and yields of suspended solids from improved temperate grassland. Journal of Environmental Monitoring: JEM 12: 731–739. 10.1039/b921584k. 20445863

[esp4398-bib-0007] Błȩdzki LA , Bubier JL , Moulton LA , Kyker‐Snowman TD . 2011 Downstream effects of beaver ponds on the water quality of New England first‐ and second‐order streams. Ecohydrology 4: 698–707. 10.1002/eco.163.

[esp4398-bib-0008] Bouwes N , Weber N , Jordan CE , Carl Saunders W , Tattam IA , Volk C , Wheaton JM , Pollock MM . 2016 Ecosystem experiment reveals benefits of natural and simulated beaver dams to a threatened population of steelhead (Oncorhynchus mykiss). Scientific Reports 6: 28581 10.1038/srep28581. 27373190 PMC4931505

[esp4398-bib-0009] Braskerud BC , Tonderski KS , Wedding B , Bakke R , Blankenberg AG , Ulén B , Koskiaho J . 2005 Can constructed wetlands reduce the diffuse phosphorus loads to eutrophic water in cold temperate regions? Journal of Environmental Quality 34: 2145 10.2134/jeq2004.0466. 16275714

[esp4398-bib-0010] Brown AG , Lespez L , Sear DA , Macaire J‐J , Houben P , Klimek K , Brazier RE , Van Oost K , Pears B . 2018 Natural vs anthropogenic streams in Europe: history, ecology and implications for restoration, river‐rewilding and riverine ecosystem services. Earth‐Science Reviews 10.1016/j.earscirev.2018.02.

[esp4398-bib-0011] Burchsted D et al. 2010 The river discontinuum: applying beaver modifications to baseline conditions for restoration of forested headwaters. BioScience 60: 908–922. 10.1525/bio.2010.60.11.7.

[esp4398-bib-0012] Burchsted D , Daniels MD . 2014 Classification of the alterations of beaver dams to headwater streams in northeastern Connecticut, USA. Geomorphology 205: 36–50. 10.1016/j.geomorph.2012.12.029.

[esp4398-bib-0013] Butler DR , Malanson GP . 1994 Beaver landforms. The Canadian Geographer/Le Géographe Canadien 38: 76–79. 10.1111/j.1541-0064.1994.tb01519.x.

[esp4398-bib-0014] Butler DR , Malanson GP . 1995 Sedimentation rates and patterns in beaver ponds in a mountain environment. Geomorphology 13: 255–269. 10.1016/0169-555X(95)00031-Y.

[esp4398-bib-0015] Butler DR , Malanson GP . 2005 The geomorphic influences of beaver dams and failures of beaver dams. Geomorphology 71: 48–60. 10.1016/j.geomorph.2004.08.016.

[esp4398-bib-0016] Chambers BJ , Garwood TWD . 2006 Monitoring of water erosion on arable farms in England and Wales. 1990–1994. Soil Use and Management 16: 93–99. 10.1111/j.1475-2743.2000.tb00181.x.

[esp4398-bib-0017] Collen P , Gibson RJ . 2000 The general ecology of beavers (Castor spp.), as related to their influence on stream ecosystems and riparian habitats, and the subsequent effects on fish – a review. Reviews in Fish Biology and Fisheries 10: 439–461. 10.1023/A:1012262217012.

[esp4398-bib-0018] Conroy J , Kitchener A . 1996 The Eurasian beaver (Castor fiber) in Scotland: a review of the literature and historical evidence. Scottish Natural Heritage Review No. 49. [online] Available from: http://www.snh.org.uk/pdfs/publications/review/049.pdf (Accessed 31st March 2015)

[esp4398-bib-0019] Correll D , Jordan T , Weller D . 2000 Beaver pond biogeochemical effects in the Maryland Coastal Plain. Biogeochemistry 49: 217–239. 10.1023/A:1006330501887.

[esp4398-bib-0021] Curran JC , Cannatelli KM . 2014 The impact of beaver dams on the morphology of a river in the eastern United States with implications for river restoration. Earth Surface Processes and Landforms 39: 1236–1244. 10.1002/esp.3576.

[esp4398-bib-0022] Deasy C et al. 2009 Mitigation options for sediment and phosphorus loss from winter‐sown arable crops. Journal of Environmental Quality 38: 2121 10.2134/jeq2009.0028. 19704154

[esp4398-bib-0023] DEFRA . 2009 Soil strategy for England. Summary: intervention and options. London. Available from: https://www.gov.uk/ (Accessed 15 June 2017)

[esp4398-bib-0024] Devito KJ , Dillon PJ , Lazerte BD . 1989 Phosphorus and nitrogen retention in five Precambrian shield wetlands. Biogeochemistry 8: 185–204. 10.1007/BF00002888.

[esp4398-bib-0025] Dillon PJ , Molot LA , Scheider WA . 1991 Phosphorus and nitrogen export from forested stream catchments in central Ontario. Journal of Environmental Quality 20: 857 10.2134/jeq1991.00472425002000040025x.

[esp4398-bib-0026] DWT . 2013 The Devon Beaver Project: The story so far. Devon Wildlife Trust [online] Available from: http://www.wildlifetrusts.org/sites/default/files/files/Beaver report 27‐8‐13.pdf (Accessed 2 February 2015)

[esp4398-bib-0027] Environment Agency . 2017 Working with Natural Processes – Evidence Directory [online] Available from: http://www.gov.uk/government/publications

[esp4398-bib-0028] Giriat D , Gorczyca E , Sobucki M . 2016 Beaver ponds’ impact on fluvial processes (Beskid Niski Mts., SE Poland). Science of the Total Environment 544: 339–353. 10.1016/j.scitotenv.2015.11.103. 26657380

[esp4398-bib-0029] Glendell M , Grangerb SJ , Bolc R , Brazier RE . 2014 Quantifying the spatial variability of soil physical and chemical properties in relation to mitigation of diffuse water pollution. Geoderma 214–215: 25–41. 10.1016/j.geoderma.2013.10.008.

[esp4398-bib-0030] Gregory AS et al. 2015 A review of the impacts of degradation threats on soil properties in the UK. Soil Use and Management 31: 1–15. 10.1111/sum.12212. 27667890 PMC5014291

[esp4398-bib-0031] Gurnell AM . 1998 The hydrogeomorphological effects of beaver dam‐building activity. Progress in Physical Geography 22: 167–189. 10.1177/030913339802200202.

[esp4398-bib-0032] Halley D , Rosell F . 2002 The beaver's reconquest of Eurasia: status, population development, and management of a conservation success. Mammal Review 32: 153–178.

[esp4398-bib-0033] Hering D , Gerhard M , Kiel E , Ehlert T , Pottgiesser T . 2001 Review study on near‐natural conditions of Central European mountain streams, with particular reference to debris and beaver dams: results of the ‘REG meeting’ 2000. Limnologica ‐ Ecology and Management of Inland Waters 31: 81–92. 10.1016/S0075-9511(01)80001-3.

[esp4398-bib-0034] Hood GA , Bayley SE . 2008 Beaver (Castor canadensis) mitigate the effects of climate on the area of open water in boreal wetlands in western Canada. Biological Conservation 141: 556–567. 10.1016/j.biocon.2007.12.003.

[esp4398-bib-0035] Hood GA , Larson DG . 2014 Ecological engineering and aquatic connectivity: a new perspective from beaver‐modified wetlands. Freshwater Biology 60: 198–208. 10.1111/fwb.12487.

[esp4398-bib-0036] Johnston CA . 1991 Sediment and nutrient retention by freshwater wetlands: effects on surface water quality. Critical Reviews in Environmental Control 21: 491–565. 10.1080/10643389109388425.

[esp4398-bib-0037] Johnston CA . 2014 Beaver pond effects on carbon storage in soils. Geoderma 213: 371–378. 10.1016/j.geoderma.2013.08.025.

[esp4398-bib-0038] Jones CG , Lawton JH , Shachak M . 1994 Organisms as ecosystem engineers. Oikos 69: 373 10.2307/3545850.

[esp4398-bib-0039] Klimenko DE , Eponchintseva DN . 2015 Experimental hydrological studies of processes of failure of beaver dams and pond draining. Biology Bulletin 42: 882–890. 10.1134/S1062359015100064.

[esp4398-bib-0040] Klotz RL . 2007 Influence of beaver ponds on the phosphorus concentration of stream water. Canadian Journal of Fisheries and Aquatic Sciences 10.1139/f97-318.

[esp4398-bib-0041] Klotz RL . 2013 Factors driving the metabolism of two north temperate ponds. Hydrobiologia 711: 9–17. 10.1007/s10750-013-1450-8.

[esp4398-bib-0042] Lamsodis R , Ulevičius A . 2012 Geomorphological effects of beaver activities in lowland drainage ditches. Zeitschrift für Geomorphologie 56: 435–458. 10.1127/0372-8854/2012/0087.

[esp4398-bib-0043] Law A , Gaywood MJ , Jones KC , Ramsay P , Willby NJ . 2017 Using ecosystem engineers as tools in habitat restoration and rewilding: beaver and wetlands. Science of the Total Environment 605: 1021–1030. 10.1016/j.scitotenv.2017.06.173. 28693107

[esp4398-bib-0044] Law A , McLean F , Willby NJ . 2016 Habitat engineering by beaver benefits aquatic biodiversity and ecosystem processes in agricultural streams. Freshwater Biology. 10.1111/fwb.12721.

[esp4398-bib-0045] Lazar JG , Addy K , Gold AJ , Groffman PM , McKinney RA , Kellogg DQ . 2015 Beaver ponds: resurgent nitrogen sinks for rural watersheds in the northeastern United States. Journal of Environmental Quality 44: 1684–1693. 10.2134/jeq2014.12.0540. 26436285

[esp4398-bib-0046] Levine R , Meyer GA . 2014 Beaver dams and channel sediment dynamics on Odell Creek, Centennial Valley, Montana, USA. Geomorphology 205: 51–64. 10.1016/j.geomorph.2013.04.035.

[esp4398-bib-0047] Lizarralde M , Deferrari G , Alvarez S , Escobar J . 1996 Effects of beaver (Castor canadensis) on the nutrient dynamics of the Southern Beech forests of Tierra del Fuego (Argentina). Ecología Austral 6: 101–105.

[esp4398-bib-0048] Macfarlane WW , Wheaton JM , Bouwes N , Jensen ML , Gilbert JT , Hough‐Snee N , Shivik JA . 2015 Modeling the capacity of riverscapes to support beaver dams. Geomorphology 10.1016/j.geomorph.2015.11.019 [online] Available from: https://www.researchgate.net/publication/285590037_Modeling_the_capacity_of_riverscapes_to_support_beaver_dams (Accessed 9 May 2016)

[esp4398-bib-0049] McKinstry MC , Caffrey P , Anderson SH . 2001 The importance of beaver to wetland habitats and waterfowl in Wyoming. Journal of the American Water Resources Association 37: 1571–1577. 10.1111/j.1752-1688.2001.tb03660.

[esp4398-bib-0050] Meentemeyer RK , Butler DR . 1999 Hydrogeomorphic Effects of beaver dams in Glacier National Park, Montana. Physical Geography 20: 436–446. 10.1080/02723646.1999.10642688.

[esp4398-bib-0051] Met_Office . 2015 Holsworthy climate information ‐ Met Office [online] Available from: http://www.metoffice.gov.uk/public/weather/climate/gchchcqqh (Accessed 2 February 2016)

[esp4398-bib-0052] Millhollon EP , Rodrigue PB , Rabb JL , Martin DF , Anderson RA , Dans DR . 2009 Designing a constructed wetland for the detention of agricultural runoff for water quality improvement. Journal of Environmental Quality 38: 2458 10.2134/jeq2008.0526. 19875802

[esp4398-bib-0053] Montgomery DR . 2007 Soil erosion and agricultural sustainability. Proceedings of the National Academy of Sciences of the United States of America 104: 13268–13272. 10.1073/pnas.0611508104. 17686990 PMC1948917

[esp4398-bib-0054] Nahlik AM , Fennessy MS . 2016 Carbon storage in US wetlands. Nature Communications 7: 13835 10.1038/ncomms13835. PMC5159918 27958272

[esp4398-bib-0055] Naiman RJ , Johnston CA , Kelley JC . 1988 Alteration of North American streams by beaver: the structure and dynamics of streams are changing as beaver recolonize their historic habitat. BioScience 38: 753–762. 10.2307/1310784.

[esp4398-bib-0056] Naiman RJ , Melillo JM , Hobbie JE . 1986 Ecosystem alteration of boreal forest streams by beaver (Castor Canadensis). Ecology 67: 1254–1269. 10.2307/1938681.

[esp4398-bib-0057] Naiman RJ , Pinay G , Johnston CA , Pastor J . 1994 Beaver influences on the long‐term biogeochemical characteristics of boreal forest drainage networks. Ecology 75: 905–921. Available from: http://doi.wiley.com/10.2307/1939415 (Accessed 11 July 2016).

[esp4398-bib-0058] Nummi P , Holopainen S . 2014 Whole‐community facilitation by beaver: ecosystem engineer increases waterbird diversity. Aquatic Conservation: Marine and Freshwater Ecosystems 24: 623–633. 10.1002/aqc.2437.

[esp4398-bib-0059] Nyssen J , Pontzeele J , Billi P . 2011 Effect of beaver dams on the hydrology of small mountain streams: example from the Chevral in the Ourthe orientale basin, Ardennes, Belgium. Journal of Hydrology 402: 92–102. 10.1016/j.jhydrol.2011.03.008.

[esp4398-bib-0060] Ockenden MC , Deasy C , Quinton JN , Bailey AP , Surridge B , Stoate C . 2012 Evaluation of field wetlands for mitigation of diffuse pollution from agriculture: sediment retention, cost and effectiveness. Environmental Science and Policy 24: 110–119. 10.1016/j.envsci.2012.06.003.

[esp4398-bib-0061] Ockenden MC , Deasy C , Quinton JN , Surridge B , Stoate C . 2014 Keeping agricultural soil out of rivers: evidence of sediment and nutrient accumulation within field wetlands in the UK. Journal of Environmental Management 135: 54–62. 10.1016/j.jenvman.2014.01.015. 24509365

[esp4398-bib-0062] Peukert S et al. 2012 Understanding spatial variability of soil properties: a key step in establishing field‐ to farm‐scale agro‐ecosystem experiments. Rapid Communications in Mass Spectrometry 26: 2413–2421. 10.1002/rcm.6336. 22976208

[esp4398-bib-0063] Peukert S et al. 2016 Spatial variation in soil properties and diffuse losses between and within grassland fields with similar short‐term management. European Journal of Soil Science 67: 386–396. 10.1111/ejss.12351. 27867311 PMC5103181

[esp4398-bib-0064] Pollock MM , Beechie TJ , Wheaton JM , Jordan CE , Bouwes N , Weber N , Volk C . 2014 Using beaver dams to restore incised stream ecosystems. BioScience 64: 279–290. 10.1093/biosci/biu036.

[esp4398-bib-0065] Polvi LE , Wohl E . 2012 The beaver meadow complex revisited ‐ the role of beavers in post‐glacial floodplain development. Earth Surface Processes and Landforms 37: 332–346. 10.1002/esp.2261.

[esp4398-bib-0066] Puttock AK , Cunliffe AM , Anderson K , Brazier RE . 2015 Aerial photography collected with a multirotor drone reveals impact of Eurasian beaver reintroduction on ecosystem structure. Journal of Unmanned Vehicle Systems 3: 123–130 150429143447007. 10.1139/juvs-2015-0005.

[esp4398-bib-0067] Puttock A , Graham HA , Cunliffe AM , Elliott M , Brazier RE . 2017 Eurasian beaver activity increases water storage, attenuates flow and mitigates diffuse pollution from intensively‐managed grasslands. Science of the Total Environment 576: 430–443. 10.1016/j.scitotenv.2016.10.122. 27792958

[esp4398-bib-0068] Quinton JN , Catt JA , Wood GA , Steer J . 2006 Soil carbon losses by water erosion: experimentation and modeling at field and national scales in the UK. Agriculture Ecosystems and Environment 112: 87–102. 10.1016/j.agee.2005.07.005.

[esp4398-bib-0069] Rolauffs P , Hering D , Lohse S . 2001 Composition, invertebrate community and productivity of a beaver dam in comparison to other stream habitat types. Hydrobiologia 459: 201–212. 10.1023/A:1012507613952.

[esp4398-bib-0070] Rosell F , Bozser O , Collen P , Parker H . 2005 Ecological impact of beavers Castor fiber and Castor canadensis and their ability to modify ecosystems. Mammal Review 35: 248–276. 10.1111/j.1365-2907.2005.00067.x.

[esp4398-bib-0071] Smith HG , Blake WH . 2014 Sediment fingerprinting in agricultural catchments: a critical re‐examination of source discrimination and data corrections. Geomorphology 204: 177–191. 10.1016/j.geomorph.2013.08.003.

[esp4398-bib-0072] Stefan J , Klein A . 2004 Hydrogeomorphic effects of beaver dams on floodplain morphology: avulsion processes and sediment fluxes in upland valley floors (Spessart, Germany). Quaternaire 15: 219–231. Available from: http://www.persee.fr/doc/quate_1142-2904_2004_num_15_1_1769 Accessed 2 June 2017.

[esp4398-bib-0073] Vecherskiy MV , Korotaeva VV , Kostina NV , Dobrovol'skaya TG , Umarov MM . 2011 Biological activities of ‘beaver landscape’ soils. Moscow University Soil Science Bulletin 66: 175–179. 10.3103/S0147687411040089.

[esp4398-bib-0074] Verheijen FGA , Jones RJA , Rickson RJ , Smith CJ . 2009 Tolerable versus actual soil erosion rates in Europe. Earth‐Science Reviews 94: 23–38 10.1016/j.earscirev.2009.02.003 2017.

[esp4398-bib-0075] de Visscher M et al. 2014 Spatio‐temporal sedimentation patterns in beaver ponds along the Chevral river, Ardennes, Belgium. Hydrological Processes 28: 1602–1615. 10.1002/hyp.9702.

[esp4398-bib-0076] Walsh SJ , Butler DR , Malanson GP . 1998 An overview of scale, pattern, process relationships in geomorphology: a remote sensing and GIS perspective. Geomorphology 21: 183–205. 10.1016/S0169-555X(97)00057-3.

[esp4398-bib-1077] Walling DE , Webb BW . 1985 Estimating the discharge of contaminants to coastal waters by rivers: Some cautionary comments. Marine Pollution Bulletin 16: 488–492. Available from: http://linkinghub.elsevier.com/retrieve/pii/0025326X85903820 (Accessed 11 July 2016).

[esp4398-bib-0077] Webb J , Loveland PJ , Chambers BJ , Mitchell R , Garwood T . 2001 The impact of modern farming practices on soil fertility and quality in England and Wales. The Journal of Agricultural Science 137 10.1017/S0021859601001290.

[esp4398-bib-0078] Wohl E . 2013 Landscape‐scale carbon storage associated with beaver dams. Geophysical Research Letters 40: 3631–3636. 10.1002/grl.50710.

